# Further Evidences for the Sulfur Spillover on Carbon: Thermodynamic Aspects and Effects on Li‐S Solid‐State Batteries

**DOI:** 10.1002/advs.76931

**Published:** 2026-08-13

**Authors:** Francesco Piccolo, Anka Berger, Sebastian Risse, Román Healy Corominas, Wilhelm Erth, Florian Fuchs, Jesus Andrade, Jörg Schuster, Marc Armbrüster, Philipp Adelhelm

**Affiliations:** ^1^ Institute of Chemistry Humboldt‐Universität zu Berlin Berlin Germany; ^2^ Research Group Operando Battery Analysis (CE‐GOBA) Helmholtz‐Zentrum Berlin für Materialien und Energie (HZB) Berlin Germany; ^3^ BAM Bundesanstalt für Materialforschung und ‐prüfung Berlin Germany; ^4^ Inorganic Solid State and Material Chemistry Eduard‐Zintl‐Institute Technical University Darmstadt Darmstadt Germany; ^5^ Chemnitz University of Technology Chemnitz Germany; ^6^ Fraunhofer Institute for Electronic Nano Systems Chemnitz Germany; ^7^ Institute of Chemistry Materials for Innovative Energy Concepts Chemnitz University of Technology Chemnitz Germany; ^8^ Center for the Science of Materials Berlin Humboldt‐Universität zu Berlin Berlin Germany

**Keywords:** activated carbon, Li‐S batteries, solid‐solid adsorption, solid‐solid wetting, sulfur spillover, sulfur, sulfur‐carbon composites

## Abstract

This study investigates sulfur spillover, the spontaneous and rapid spread of sulfur over carbon surfaces at ambient temperature. The process is studied by small‐angle X‐ray scattering (SAXS), X‐ray radiography, and microcalorimetry from which a number of physicochemical properties are derived. The results confirm that sulfur immediately interacts with porous carbon upon mixing, with most signal changes occurring within a few hours. For the first time, thermodynamic data of the process are determined. Using microcalorimetry, the lower bound of the reaction enthalpy is determined to be Δ_sp_ *H* = −28.2 kJ mol_S8_^−1^. With an estimated reaction entropy of Δ_sp_ *S* = 273.78 J mol_S8_^−1^ K^−1^, the Gibbs Free Energy of the spillover process is estimated as Δ_sp_ *G* = −100.23 kJ mol_S8_^−1^, which reduces the theoretical cell voltage of Li‐S batteries by about 64 mV. Furthermore, depending on the configuration, DFT calculations have revealed repulsive and attractive interactions; the latter are caused by defects that eventually result in ring opening and the formation of S─C bonds. This suggests that spillover includes various processes that occur simultaneously. Considering metal‐sulfur batteries, sulfur spillover may be critical for preparing and cycling sulfur‐carbon composite cathodes, which is demonstrated for a Li–S solid‐state battery.

## Graphical abstract

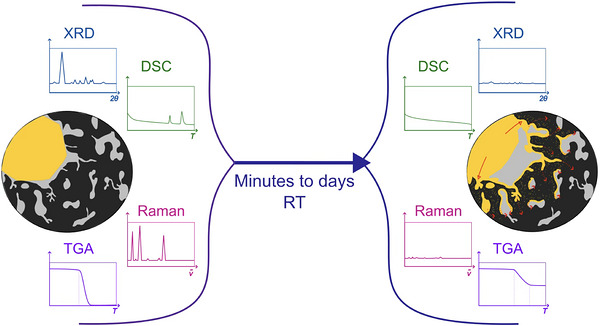

## 1 Introduction

**FIGURE 1:**
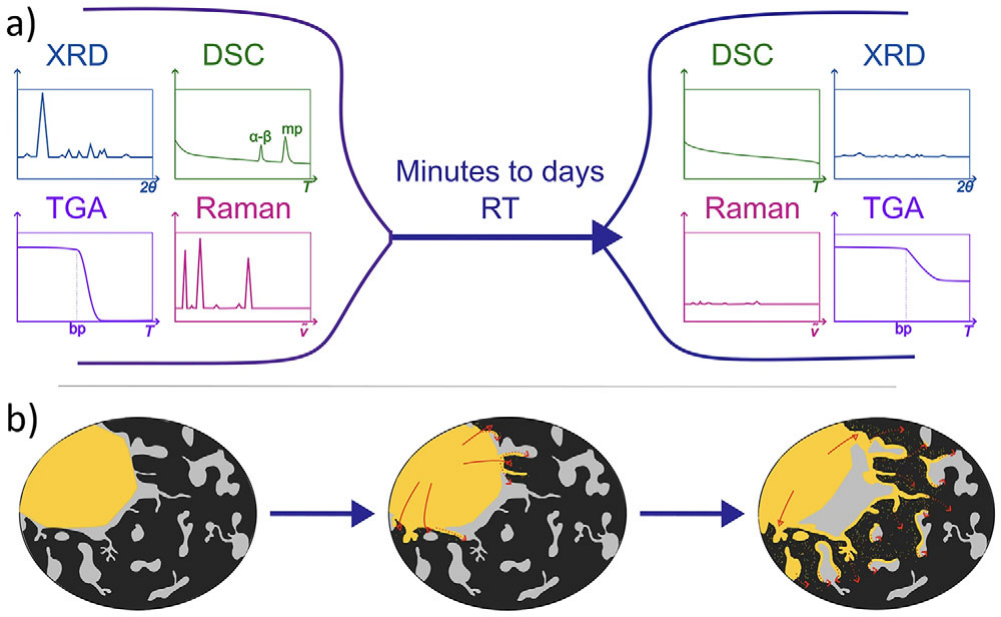
(a) Loss of sulfur bulk properties induced by sulfur spillover onto carbon: loss of sulfur crystallinity (XRD), loss of sulfur α→β transition and melting point (DSC), higher sulfur evaporation temperature (TGA), loss of sulfur vibrational modes (Raman spectroscopy); (b) Illustration of the sulfur spillover: The process is characterized by the continuous spreading of sulfur over the surface and into the pores of the activated carbon, accompanied by the gradual loss of the bulk properties of sulfur.

The chemistry of sulfur is generally known to be complex due to a large number of polymorphs and allotropes [1]. Recently, it was found that sulfur loses its bulk properties if brought in contact with carbon materials with high surface area. This so‐called “sulfur spillover” effect was first reported by Raiß et al. [2], and later studied by Medenbach et al. [3], as well as Pal and Bhattacharyya [4] and proven for several different activated carbons. After simply mixing sulfur and carbon in a mortar, the bulk properties of sulfur such as its crystallinity (as detected by X‐ray diffraction [XRD]), melting point (as determined by differential scanning calorimetry [DSC]), and molecular vibrations (as monitored by Raman spectroscopy), gradually disappear for samples that are stored at room temperature for a few days (Figure 1a). It is believed that such a process is caused by the progressive covering of carbon surfaces along with the filling of its pores, as the BET surface area is found to significantly decrease at the same time (Figure 1b) [2].

The amount of sulfur that can undergo spillover is limited by the properties of the carbon material. Each carbon material is characterized by an intrinsic “spillover capacity”, defined as the maximum amount of sulfur that can undergo spillover. The spillover capacity is expressed as the mass ratio of sulfur to carbon (*m*_S_/*m*_C_), depends upon the surface area of the carbon and can easily exceed 50 wt.% for highly porous materials [3]. For sulfur loadings below this capacity, all sulfur loses its bulk properties. For values exceeding it, some of the sulfur remains in its bulk state, showing the typical bulk properties such as a defined melting point, crystallinity, and so forth. Medenbach et al. studied a variety of sulfur/carbon mixtures and calculated for a carbon with a surface area of 2070 m^2^ g^−1^ a spillover capacity of 1.54 g_S_ g_C_^−1^ [3]. Pal and Bhattacharyya investigated the phenomenon for four different activated carbons with surface areas ranging from 979 m^2^ g^−1^ to 2931 m^2^ g^−1^ [4]. All samples contained the same sulfur loading of 50 wt.%. For carbon materials with surface areas exceeding 2357 m^2^ g^−1^, a complete spillover was observed, i.e., all bulk properties were lost. For those with surface areas of 1809 m^2^ g^−1^ or lower, some bulk sulfur remained in the sample, indicating that the spillover capacity was exceeded. The surface area alone, however, is likely not the only factor determining the spillover capacity as also other effects such as the morphology (pore distribution and shape) and the surface structure and impurities in the carbon could affect the interactions with sulfur. To assess the influence of the chemical composition of the support, Medenbach et al. conducted experiments with high surface area silica for which no spillover could be observed [3]. This suggests that the occurrence of the sulfur spillover may require specific interactions between sulfur and the porous material.

Overall, although the spillover process is very evident from experiments, a clear explanation of its causes and underlying principles is still missing. To get a better understanding, similarities with the more often observed physical phenomenon of “solid‐solid adsorption” [5] or “solid‐solid wetting” [6], by which a solid spontaneously spreads over a porous support at temperatures below its melting point, are discussed. Xie Youchang et al. were the first to systematically investigate this effect for several combinations of materials [5]. While they mostly focused on oxides and halides over oxide supports [5, 7], they also briefly reported on activated carbon as a support (with HgCl_2_ and ZnAc_2_ as adsorbed species) [7], and sulfur as an adsorbed species (with γ‐Al_2_O_3_ as support) [8]. The authors suggested that the driving force for this process is the formation of strong surface bonds between the adsorbed species and the support and the total entropy increase when transitioning from an ordered three‐dimensional crystal (before wetting) to a less‐ordered two‐dimensional phase (after wetting).

Expanding on this, Knözinger and Taglauer described this process in terms of surface and interface free energies between the phases and highlighted its analogies with the wetting of a solid by a liquid; hence, they named the process “solid‐solid wetting” [6]. As in the liquid case, a solid wetting is associated with the formation of a large interface between the adsorbed species and the support and between the adsorbed species and the surrounding gas phase, while at the same time the interface between the support and the gas phase becomes smaller. If this change reduces the overall energy of the system, then the process is thermodynamically favorable. However, such a process can, according to Knözinger and Taglauer, only happen above the Tammann temperature of the adsorbed species. The Tammann temperature of a non‐metallic compound (i.e., oxides) is roughly 2/3 of *T_m_* and corresponds to the temperature at which the atoms, ions, or molecules in the bulk are sufficiently mobile for bulk‐to‐surface migration [9]. A related aspect is the well‐known liquid‐like characteristics of solids surfaces at temperatures significantly below the melting point as extensively studied first by Hüttig [10, 11].

Finally, an extensive discussion on the thermodynamic origin of solid‐solid wetting was provided by Kaplan et al., who included factors such as the contact angle, the anisotropy of crystals, and the de‐wetting processes [12]. These aspects are, however, difficult to generalize across carbon materials, as the term “carbon materials” encompasses a broad class of solids that can differ markedly in structure, surface chemistry, and elemental composition. Nevertheless, there appears to be good agreement between observations related to solid–solid wetting (e.g., as evidenced by XRD and DSC) and the S–C spillover effect reported by Medenbach et al. [3].

Considering sulfur as an adsorbate, it is important to recall that it consists of S_8_ rings held together by relatively weak van der Waals forces, resulting in a lattice energy of 106 kJ mol^−1^ (α‐S_8(solid)_ to S_8(gaseous)_ at 25°C) [2]. This explains the low melting point (*T_m_* = 115°C), from which a Tammann temperature of −10°C results, suggesting a significant mobility of the S_8_ molecules at ambient temperature, thus enabling a solid‐solid wetting.

Overall, different terminologies have been used in the past to describe solid‐solid interactions (“solid–solid adsorption” [5], “solid–solid wetting” [6] and “sulfur spillover” [3]) that cause a loss in bulk properties of the adsorbate. The underlying physical phenomena appear to be closely related, so they may be grouped into the same category of solid‐solid interactions. To avoid confusion, in the following the term “spillover” is used to highlight the connection with the previous studies on the sulfur‐carbon system.

The spillover process is not just a scientific curiosity, but may have far‐reaching consequences for the development of metal‐sulfur batteries, as already indicated by Medenbach et al. [3]. Indeed, sulfur‐carbon composites have attracted considerable interest over the years as possible cathode materials for metal‐sulfur batteries [13, 14]. Here, the spillover process may lead, e.g., to a redistribution of sulfur in batteries when being charged or when stored in the charged state. The process could also simplify the fabrication of sulfur‐carbon composites as a sufficiently long storage time will lead to pore filling and close contact between sulfur and carbon, this way improving the electrochemical activity [15].

So far, the spillover process of sulfur and carbon has been studied by X‐ray diffraction (XRD) [3, 4], differential scanning calorimetry (DSC) [3, 4], Raman spectroscopy [3, 4], N_2_ physisorption (BET) [2, 4], thermogravimetric analysis (TGA) [3, 4], scanning electron microscopy/energy dispersive X‐ray spectroscopy (SEM/EDX) [2, 3], electron paramagnetic resonance spectroscopy (EPR) [4], UV–vis spectroscopy [4], and galvanostatic cycling [3, 4]. To learn more about the phenomenon, the spillover process is here studied for the first time with several more methods: small angle X‐ray scattering (SAXS), to study which pores are filled by sulfur and how fast the process is; X‐ray radiography, to follow the spreading of sulfur over the carbon support on a macroscopic scale; microcalorimetry, to estimate the reaction enthalpy and, indirectly, the Gibbs free energy and the effect on a cell voltage; DFT, to reveal possible chemical interactions. Finally, solid‐state Li‐S batteries were prepared with a focus on the spillover effect to explore the effect's relevance for batteries.

## 2 Results and Discussion

The effect was explored for sulfur‐carbon (S:C) mixtures with different ratios of the two components. The mixtures were prepared by gently blending sulfur powder and activated carbon using a hand mortar. The resulting powders were collected and analyzed. For all measurements, high surface area carbons were used.

### 2.1 Small‐Angle X‐Ray Scattering (SAXS)

**FIGURE 2:**
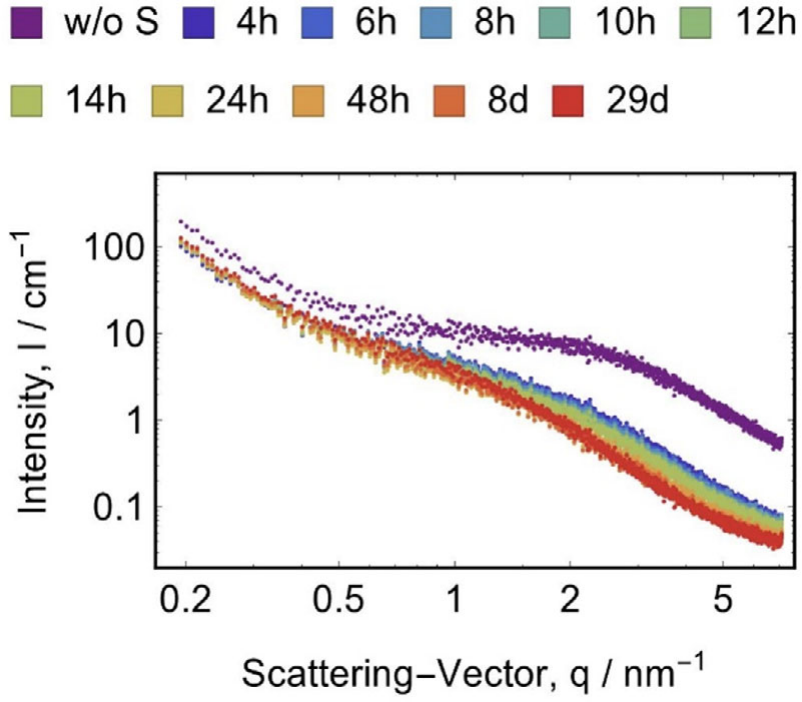
SAXS of pristine activated carbon (w/o S) and sulfur–carbon composite powders measured at various time intervals after mixing, ranging from 4 h to 29 days.

Small‐angle X‐ray scattering (SAXS) is particularly well‐suited for studying the interaction between elemental sulfur and activated carbon, as it provides sensitive insights into the wetting and infiltration of the carbon microstructure by sulfur. Sulfur and carbon exhibit very similar scattering length densities; therefore, the filling of micropores manifests as a pronounced decrease in scattering intensity within the range characteristic of pore scattering (approximately 1–5 nm^−1^) [16]. Figure 2 displays SAXS recorded at various time intervals, ranging from 4 h to 29 days, following the preparation of the sulfur–carbon powder mixture. Compared to the scattering profile of pristine activated carbon (without sulfur), a distinct reduction in intensity is observed in the range of 1–5 nm^−1^. This indicates that, even at ambient temperature, a substantial portion of the sulfur penetrates the carbon pores within 24 h, and this infiltrated state remains stable over at least 29 days.

**FIGURE 3:**
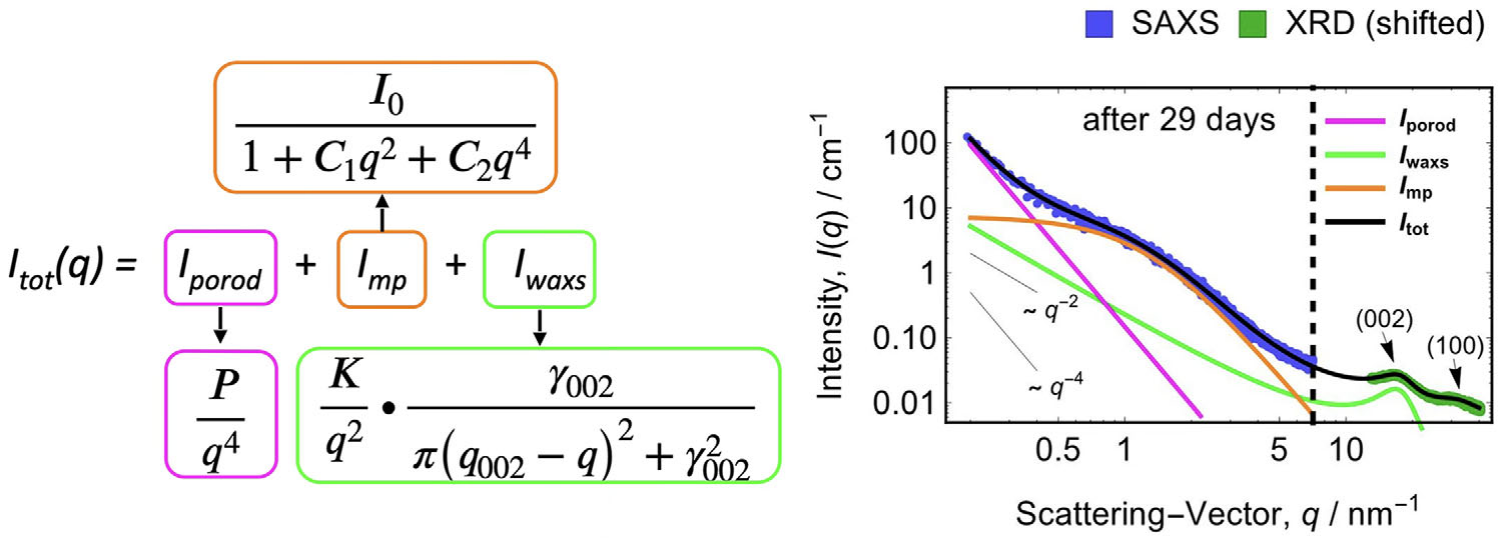
Left: Principal contributions to the intensity profile and relative intensity functions. Right: Extended SAXS of the carbon/sulfur sample after 29 days. The individual contributions according to the equation *I_tot_* (*q*)=*I_porod_*  +*I_mp_* +*I_waxs_* are shown.

To analyze the SAXS of pristine and sulfur‐loaded activated carbon, the intensity (in cm^−1^) was modeled as the sum of three principal contributions (Figure 3): Porod scattering (*I_porod_*), due to sharp interfaces from macroscopic surface roughness; Teubner–Strey scattering (*I_mp_*), describing the microporous disordered network, and broad wide angle X‐ray scattering (WAXS) features (*I_waxs_*), notably two diffuse scattering maxima observed at *q_p_ =* 17.6 and 32.9 nm^−1^ corresponding to the (002) and (100) reflections of carbon.

Once the three terms are resolved to their respective intensity functions, the final model contains only five adjustable parameters, *P*, *I_0_*, *C_1_*, *C_2_* and *K*, to describe all 11 SAXS curves recorded between 0 h and 29 days after mixing. Figure S1 presents a summary of the resulting fit across all time points.

**FIGURE 4:**
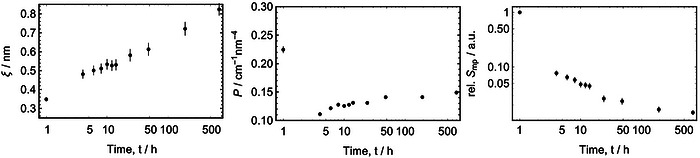
Evolution of structural parameters extracted from fitting of the SAXS data over time after sulfur infiltration. Left: Correlation length ξ obtained from the Teubner–Strey model. Center: Porod constant, reflecting the total internal surface area of sharp interfaces. Right: Relative micropore surface area rel. *S_mp_*, normalized to the pristine activated carbon. All parameters indicate progressive pore filling by sulfur over the course of 29 days with most of the changes occurring within the first 24 h.

Figure 4 summarizes the calculated correlation length (ξ), Porod constant (*P*) and relative micropore surface area (rel. *S_mp_*), obtained from the fit parameters (more details in the Supporting Information). In the Teubner–Strey model, ξ describes the average spatial extent of short‐range ordering between two distinct phases – here, the carbon matrix and the empty pore volume. The increase in the correlation length ξ from 0.35 nm in pristine activated carbon to 0.8 nm after 29 days of sulfur exposure can be interpreted as a consequence of progressive micropore filling by sulfur.

A low ξ in the pristine sample reflects the dense, fine‐scale heterogeneity of the microporous structure, where numerous carbon‐void interfaces exist within short distances. As elemental sulfur diffuses into and wets the internal pore surfaces, it effectively replaces the pore voids, thereby eliminating electron density contrast between the two phases. Because sulfur and carbon have nearly identical scattering length densities, the scattering contribution from the filled pores vanishes. This leads to an apparent increase in ξ, not because the actual structural correlation grows, but because the measurable heterogeneity becomes more spatially uniform from the SAXS perspective. Two key observations further reinforce this interpretation. First, the Porod constant, *P*, drops sharply, indicating a significant loss of internal surface area as the well‐defined interfaces between carbon and pore voids disappear. Second, the relative micropore surface area rel. *S_mp_* decreases dramatically from 1.0 in the pristine material to just 0.03 after 24 h of sulfur exposure, confirming that the majority of the microporous surface becomes effectively invisible to SAXS within the first day, consistent with rapid and nearly complete pore filling.

Taken together, the increase in correlation length ξ, the reduction in Porod scattering, and the vanishing micropore surface signal all provide coherent and compelling evidence for efficient sulfur infiltration into the microporous network of the activated carbon, most of which takes place within the first 24 h.

### 2.2 X‐Ray Imaging

**FIGURE 5:**
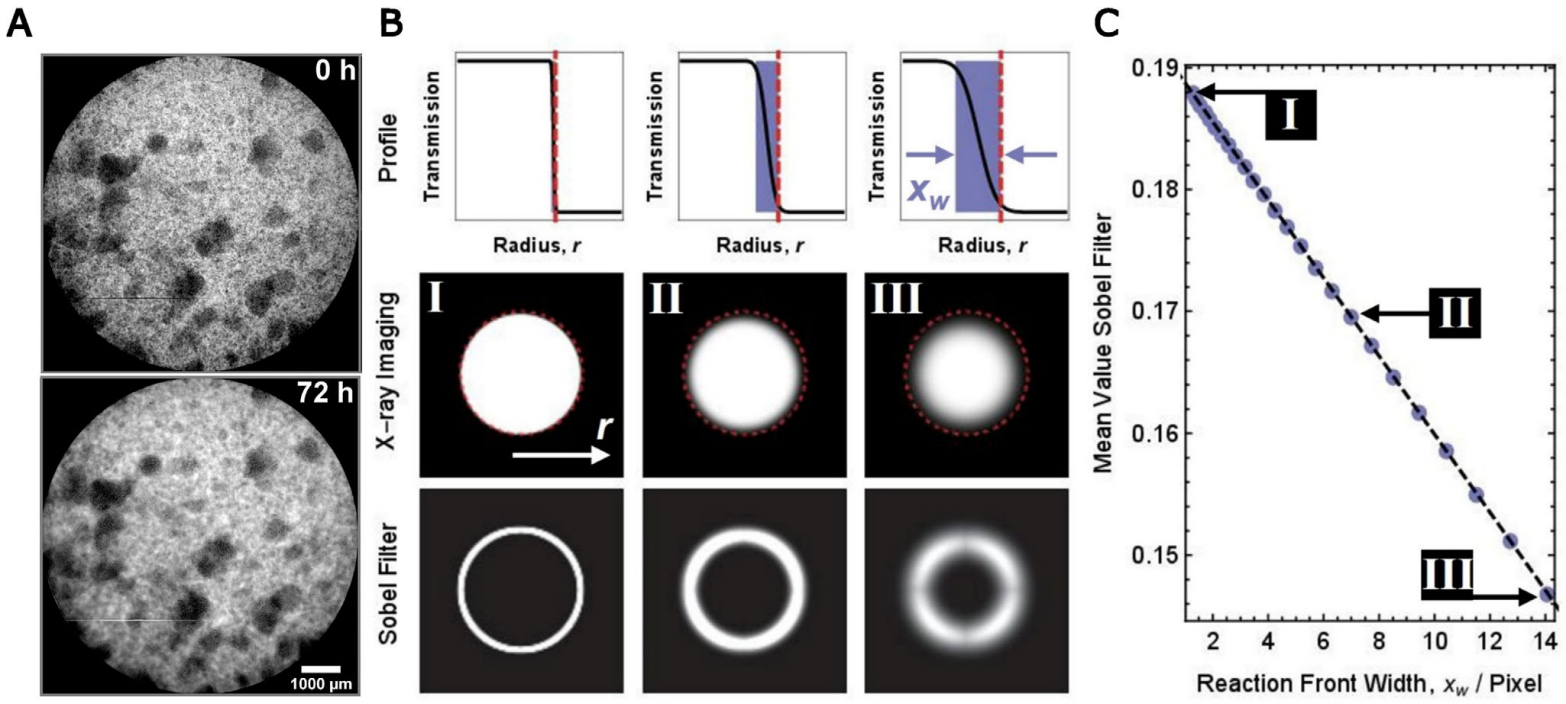
(A) Comparison of radiography images of the S:C mixture in the airtight cell. Top: Freshly prepared S:C mixture with sulfur particles (dark) and carbon powder background (bright). Bottom: Radiography image of the same sample after 72 h. The contrast was decreased due to S:C phase mixing on the macroscopic scale. (B) Upper row: Calculated transmission profile of a carbon particle during the in‐diffusion of sulfur. Middle row: Simulated X‐ray image of an idealized particle that fulfils the respective transmission profile. Lower row: Processed images where the Sobel Filter was applied. (C) Linear relationship between mean grey value of the Sobel filter and the reaction front.

X‐ray radiography was used to investigate the interaction of sulfur with the carbon support on a macroscopic scale, as sulfur with 16 electrons absorbs significantly more X‐rays than carbon with only 6 electrons. Figure 5A shows two radiograph images taken shortly after the preparation of the S:C powder mixture and after 72 h. It can be clearly seen that the contrast between the dark sulfur particles and the bright carbon has diminished, caused by the spread of the sulfur over the carbon during the three days of contact.

A more detailed data analysis can be applied to elucidate the nature of the apparent mixing process of carbon and sulfur powder in Figure 5. Figure 5B displays a model carbon particle (white) that is in contact with sulfur (black). If the filling process is determined by a diffusion process, the sulfur concentration *c*(*r*, *t*) in the carbon particle would follow in a first and simple approximation $c\;\left(r,t\right)=c_{0}\;Erfc\left[\frac{r}{2\sqrt{Dt}}\right]$ [17]. Here, *r* is the radius, *D* the diffusion constant, *c_0_* the initial sulfur concentration outside of the particle, and *t* the time of the experiment. One consequence of this model, where sulfur successively fills the porous carbon structure, is that the reaction front width, *x_w_*, is proportional to $\sqrt{Dt}$. A Sobel filter can be applied to the simulated X‐ray image with the respective diffusion profile, so that the edges of the particle can be visualized. If one measures the mean grey value of the complete Sobel filter image obtained, one finds that there is a linear relationship between both values.

**FIGURE 6:**
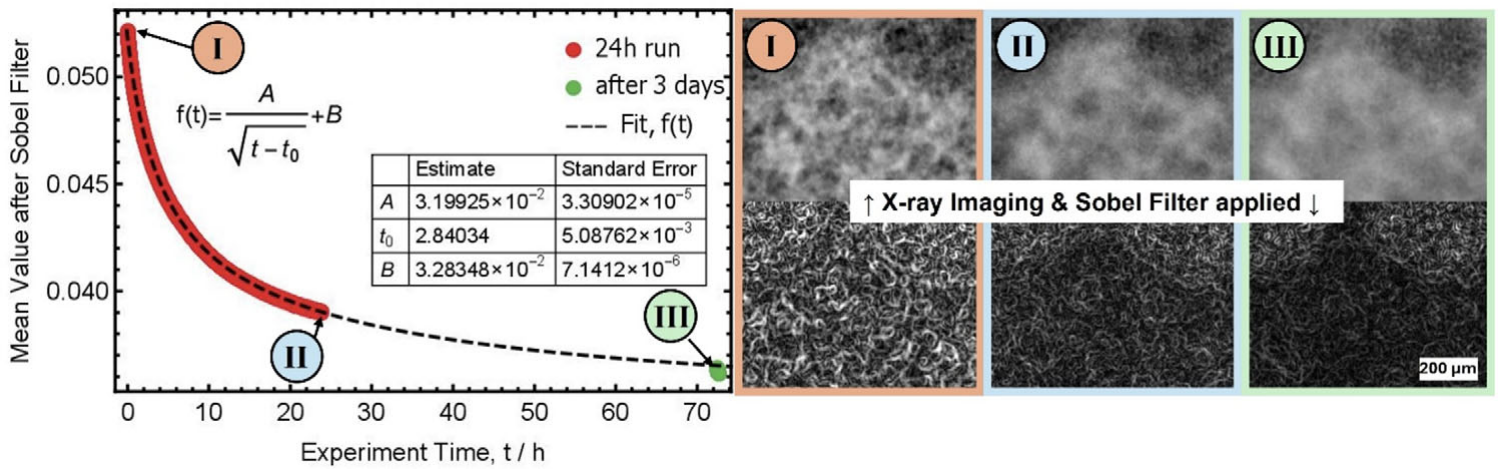
Comparison of radiography images of the S:C mixture in the operando cell. Left: Freshly prepared S:C mixture with dark remaining sulfur (Z = 16) particles and bright carbon (Z = 6) powder background yielding an overall operando cell transmission of 67.9%. Right: Radiography image of the very same sample after 72 h with an overall transmission of 75.5%. The contrast was decreased due to S:C phase mixing on the macroscopic scale.

If the process is diffusion‐limited, the decay of the Sobel filter mean grey value should follow a slope that is proportional to $f\;\left(t\right)=A{\left(t-t_{0}\right)}^{-\frac{1}{2}}+B$. Here, *A* is a fitting constant that includes proportionality constants as well as the diffusion constant, *t_0_* represents an offset in time, and *B* displays a limit for the contrast between filled carbon and sulfur. This can be used to analyze the time‐dependent X‐ray images of the actual experiment. Figure 6 shows the result of this fit and the respective X‐ray images with their Sobel filter counterparts. Although the fitting function *f(t)* was only fitted to the data set that was obtained in the first 24 h (red), the curve can still predict the state of the powder mixture after 3 days of the experiment (green).

All three fitting parameters show a very small standard error of less than 1%, which indicates a strong fitting quality for this diffusion‐limited model. Exponential fits or the fit to different kinetic orders were not successful. From the X‐ray images I‐III in Figure 6, the contrast between the individual particles vanishes over time. The same effect can also be seen in the respective images, where the Sobel filter was applied. In the initial phase, edges are very pronounced and start fading with ongoing experimental time.

### 2.3 Microcalorimetry and the Enthalpy of the Reaction

**FIGURE 7:**
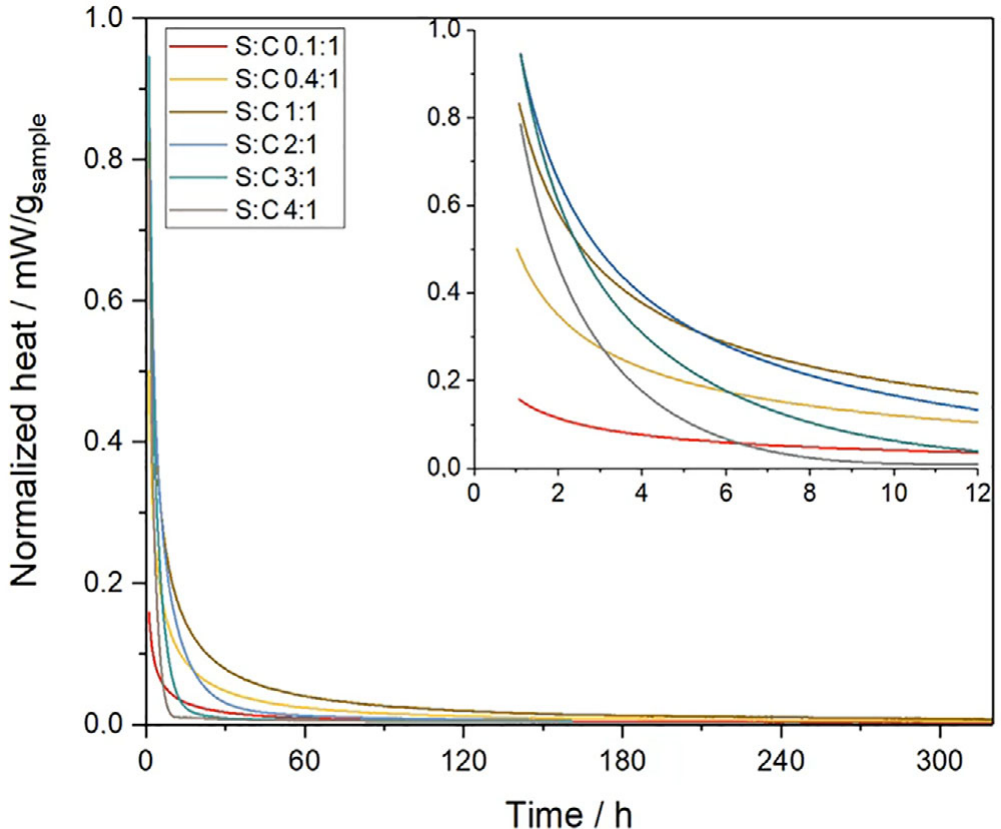
Heat evolution of mixtures with different S:C ratios at 25°C. The data were normalized according to the total weight of the sample (250 mg for ratios from 0.1:1 to 1:1, 400 mg for ratios from 1.5:1 to 4:1). The measurements started approximately 1 h after the preparation of the mixtures (*t* = 0).

Microcalorimetry has been employed to quantify the enthalpy of the spillover process. Sulfur‐carbon mixtures of different ratios were prepared and inserted into the microcalorimeter, which monitored the heat release over several days at 25°C ((Figure 7) and Figure S2). It is important to note that the time between the insertion of the samples in the instrument and the start of the measurement was kept to a minimum (∼20 min).

A well‐defined exothermic signal appeared just a few minutes after the start of the measurement (∼1 h after mixing). This very fast kinetic response at room temperature (with >75% of all the heat released over 12 days concentrated in the first three days and ∼10% in the first hour) is a challenge for the quantification of the calorimetry data, as the heat signal likely peaks already during mixing of the samples. The experimentally determined enthalpy values should therefore be seen as a lower limit, i.e., the true values will be larger. On the other hand, it is worth noting that the method allows to study larger amounts of sample, which generally helps to derive accurate values.

A series of reference experiments was made to confirm that the measured enthalpy is due to the spillover effect. The possibility that the signal was caused by adsorption or reaction of other species such as water on carbon (which had been pre‐dried under vacuum) or on sulfur, or that it was caused by the mixing step, was ruled out by measuring reference samples of just the carbon (pristine or mortared for 2 min) or sulfur (Figure S3). None of them released any significant heat, confirming that the heat measured corresponds to the interaction of sulfur and carbon. The reproducibility of the results was also confirmed with specific tests (Figure S4).

The possibility that the reaction was caused by the high surface area of the support alone or by the carbon chemical properties alone (like in a bulk reaction) was ruled out as well by comparing activated carbon, zeolite (no carbon but high surface area (600–700 m^2^ g^−1^)) and carbon nanofibers (carbon but low surface area (24 m^2^ g^−1^)), all in a S:X 2:1 ratio and at 60°C (Figure S5). While the zeolites employed have a surface area between 1/3 and 1/2 compared to the activated carbon, the heat released upon mixing with sulfur is only ∼1/25. For the carbon nanofibers sample, no relevant heat release could be detected. However, it should be noted that the pore diameter of the zeolite used was 5 Å, which is only slightly larger than the diameter of an S_8_ molecule (∼4.1 Å) [18]. It therefore cannot be entirely excluded that spillover was limited due to the small pore size.

**FIGURE 8:**
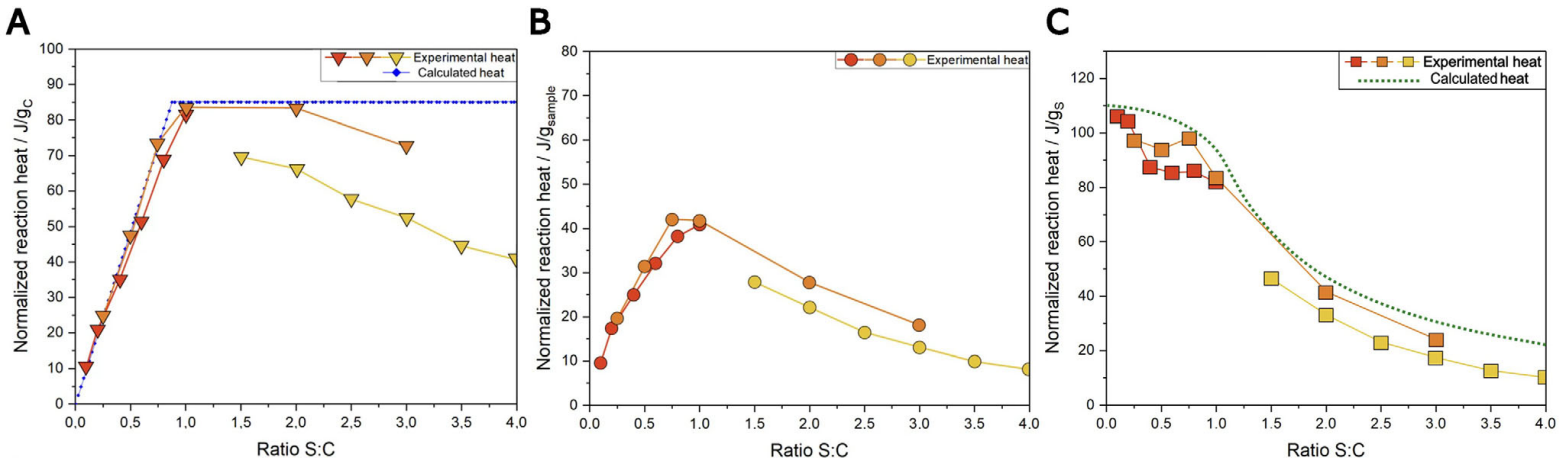
Cumulative heat release of mixtures with different S:C ratios at 25°C normalized for carbon weight (A), total sample weight (B) and sulfur weight (C). The three different colours correspond to the three different sets of measurements. Each measurement proceeded until the heat evolution reached a value of about ∼1 µW. A) The blue dots correspond to the calculated heat release at the different ratios assuming there is only one kind of interaction. B) The maximum of the curve corresponds approximately to the sulfur spillover capacity of the carbon. C) The green dotted line indicates the calculated heat evolution assuming a finite number of heterogeneous adsorption sites (the last ones to be occupied will be less energetically favourable).

The influence of the mixing time was also investigated (Figure S6). Three samples of S:C 1:1 were mixed respectively for 1, 2, and 4 min. While increasing the mixing time improves the homogeneity of the sample, the deviations between their heat signals are minimal, indicating the effect proceeds similarly once a threshold dispersion is produced. The cumulative heat (corresponding to the integrated heat release) of the reactions in the different measurements are combined in Figure 8. As mentioned above, it is important to realize that there is always an experimental gap due to the time needed to prepare the sample, load it in the instrument, and start the measurement. As a result, some of the heat release is not detected, and the determined values should therefore be seen as a lower estimate. Moreover, the differences in exact measuring times caused some differences between the different experimental series.

As can be seen, the heat release first increases with increasing sulfur content. Theoretically, this increase should end once the complete carbon surface is wetted, i.e., the spillover capacity is reached. Sulfur that is added beyond that point will remain in its bulk state and will not interact with the carbon support. In this case, carbon is reaction‐limiting, and the carbon‐specific enthalpy should be constant (blue plateau in Figure 8A). However, the calorimetric data reveal a decrease for ratios higher than 1.5:1. This can either mean that the C‐S interaction decreases with increasing sulfur content, or that this is caused by not detecting an increasingly higher portion of the enthalpy because of an increasing reaction speed. That the latter might be the case is indicated by the behavior of the different mixtures in Figure 8A, where the heat is released the sooner and the steeper the higher the S:C ratio is. Decreasing the S:C ratio to less than 1 leads to a linear decrease, indicating that the sulfur becomes reaction‐limiting. By plotting the weight‐specific reaction heat, the spillover capacity can be determined by the obtained maximum (Figure 8B).

In this case, the maximum lies between a S:C ratio of 0.75 and 1, corresponding to 0.464–0.619 mg_S_ m^−2^. Inserting these values and the surface area of the carbon (1616 m^2^ g^−1^) into the equation previously developed by Medenbach et al. [3],

$$L_{S,max}=\frac{m_{S}}{m_{C}}=\frac{PA_{BET}M_{S}}{\pi r_{S}^{2}N_{A}}=1.566\times 10^{-3}gm^{-2}\cdot PA_{BET}$$

where *L*_S, max_ is the spillover capacity, *A*_BET_ the carbon‐specific surface area, *M*_S_ the molar mass of sulfur, *r*_S_ its atomic radius, and *N*_A_ Avogadro's number, it is possible to estimate the degree of surface coverage *P* as being between 0.30 and 0.40. This is a purely geometrical approach assuming a monolayer of sulfur atoms (hard spheres) with radius r = 1.04∙10^10^ m and an ideally flat surface of the carbon [3]. The resulting value is slightly lower, but in the same range compared to the value reported by Medenbach et al., (0.47) that was determined using XRD and a different high surface area carbon [3].

At very low sulfur concentrations, it can be assumed that all the sulfur interacts with carbon, i.e., sulfur is reaction‐limiting. This is reflected by the emergence of a shoulder when the heat is normalized against the mass of sulfur (Figure 8C), which reaches a value of approximately 110 J g^−1^, corresponding to an enthalpy change of −28.2 kJ mol_S8_^−1^ at 25°C. This is a relatively small value compared to, for example, the calculated adsorption energy of an isolated S_8_ ring on graphene (∼60 kJ mol_S8_^−1^) [19]. However, the calorimetric experiment likely underestimates the enthalpy change (see Discussion above), and the measured value represents the sum of multiple enthalpic contributions, which may include endothermic terms depending on the nature of the sulfur–carbon interaction, such as bond breaking.

### 2.4 Entropy and Gibb´s Free Energy Estimation of the Spillover Process

An estimate for the Gibbs free energy of the spillover process (Δ_sp_ *G*) can be calculated from the measured enthalpy (Δ*H*) and the entropy change (Δ*S*). The entropy change cannot be measured directly, but can be estimated mathematically by treating it like a general case of physisorption. The entropy of an adsorbed phase can be derived from the entropy of the same compound in the gas phase with the formula *S*_ads_° = 0.7 *S*_gas_° – 3.3R, where R is the gas constant (8.314463 J mol^−1^ K^−1^) [20]. This value corresponds to roughly 2/3 of the entropy of the gas under the same conditions, also interpretable conceptually as the same gas losing one translational degree of freedom. This means that physisorption for a gas is always associated with a loss of entropy. From this formula and the standard molar entropy of an S_8_ gas [21], we can calculate the entropy of S_8_ when adsorbed on carbon, which amounts to 273.78 J mol^−1^ K^−1^. The entropy change of the process can then be calculated as the difference between this value and the standard molar entropy of the crystalline phase (*S*_S8_°_rhombhohedral_ = 32.06 J mol^−1^ K^−1^) [21]. This amounts to 241.72 J mol^−1^ K^−1^, indicating that the entropy of the system actually increases significantly with the spillover. This is an upper limit, since the small porosities may partially limit the translational, rotational, and vibrational motions of the S_8_ rings with the result of lowering the entropy [22]. Note that the estimate also neglects further entropic contributions that may occur through more specific interactions between sulfur species and the carbon surface.

Substituting the calculated values into the equation Δ_sp_ *G* = Δ*H* – TΔ*S*, the Gibbs free energy for the reaction can be obtained as follows:

$$\begin{array}{cc} \Delta_{sp}G & =\left(-28.2kJmol_{S8}^{-1}\right)\;-\left(298K\right)\;\left(241.72Jmol_{S8}^{-1}K^{-1}\right)\\ & \;=-100.23kJmol_{S8}^{-1} \end{array}$$

The negative Gibb´s free energy value confirms that the reaction is strongly thermodynamically favored. It is also important to note that the entropy term (TΔ*S*) accounts for almost ¾ of the total energy of the reaction, which can then be defined as entropy‐driven. This is similar to the solid‐solid adsorption as suggested by Xie [5] and Knözinger [6].

**FIGURE 9:**
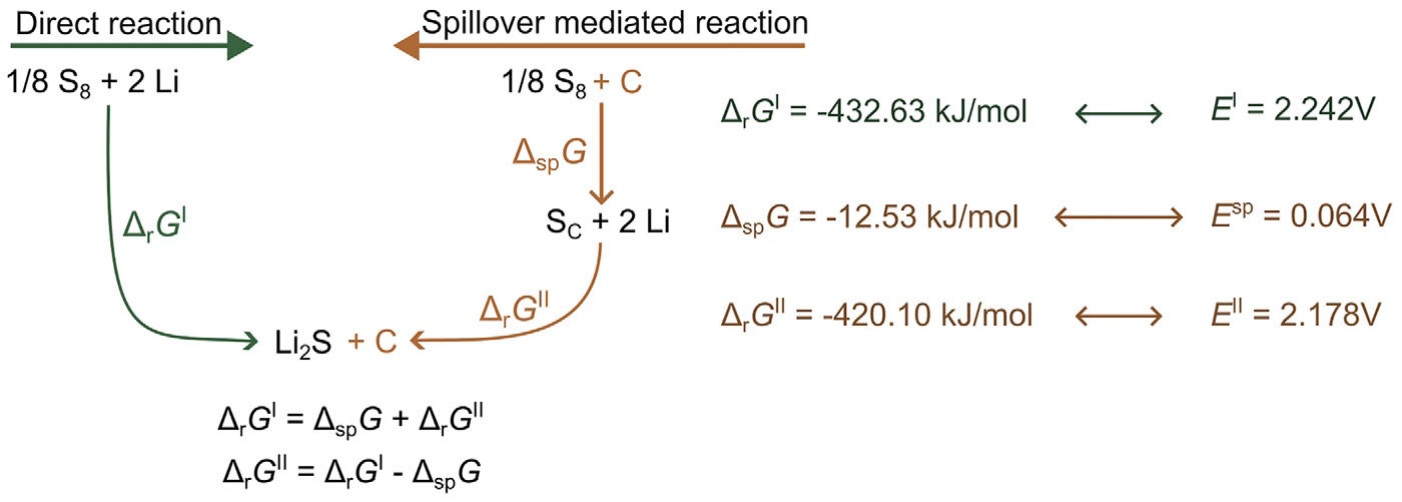
Gibbs free energy and related potential changes in a Li‐S battery at 25°C. Since the Gibbs free energy is a state function, the total Gibbs free energy change for the overall process is independent of the path taken. Therefore, the sum of the Gibbs free energy changes for the two‐step process (Δ_sp_G and Δ_r_G^II^ (spillover‐mediated) must equal the Gibbs free energy change for the direct process. The theoretical potential for the reaction with the non‐adsorbed sulfur was taken from literature [23, 24, 25] and confirmed with the software HSC Chemistry [26].

Despite some uncertainty in the quantification of the data, it is clear that there is an attractive net interaction or reaction between sulfur and carbon. This changes the thermodynamics of the ideal cell reaction for a Li‐S battery, effectively leading to a lowering of the theoretical cell voltage *E*, as shown in Figure 9. The figure compares values for *E* with and without sulfur spillover using the thermodynamic relationship Δ*G* = –z*FE*. For the reaction between Li and S_8_ to form Li_2_S (cell reaction 2 Li + 1/8 S_8_ → Li_2_S), *E* equals 2.242 V. For the reaction between Li and sulfur that has undergone the spillover process (S_sp_) to form Li_2_S, *E* equals 2.178 V. This means the cell voltage for a Li‐S battery should decrease by approximately 64 mV, a value that falls well within the typical open circuit voltages of Li‐S batteries.

### 2.5 Density Functional Theory Calculations

In addition to the experimental studies and the entropy estimate, density functional theory (DFT) calculations were made in order to rationalize the most basic interaction between sulfur and carbon. Note that the real structure of activated carbon and the S_8_ molecule is complicated and consists of many different local arrangements on the atomic scale, which would require significant simulation efforts to sample all structures. Since the goal is a first estimate, the focus is on a few simplified structures. The starting system consisted of a relaxed S_8_ molecule in close proximity (1 Å) to a relaxed rounded graphene flake passivated with hydrogen atoms on the edge. The S_8_ molecule was first centered above the graphene flake in the following positions: above a carbon atom, above a carbon–carbon bond, and above the center of a C_6_ hexagon. After optimization, no reaction was observed between carbon and sulfur, instead the S_8_ molecule was repulsed by the graphene flake to a distance between 3–4 Å. Moreover, upon optimization, the centering over the atom transformed into a centering over a bond.

**FIGURE 10:**
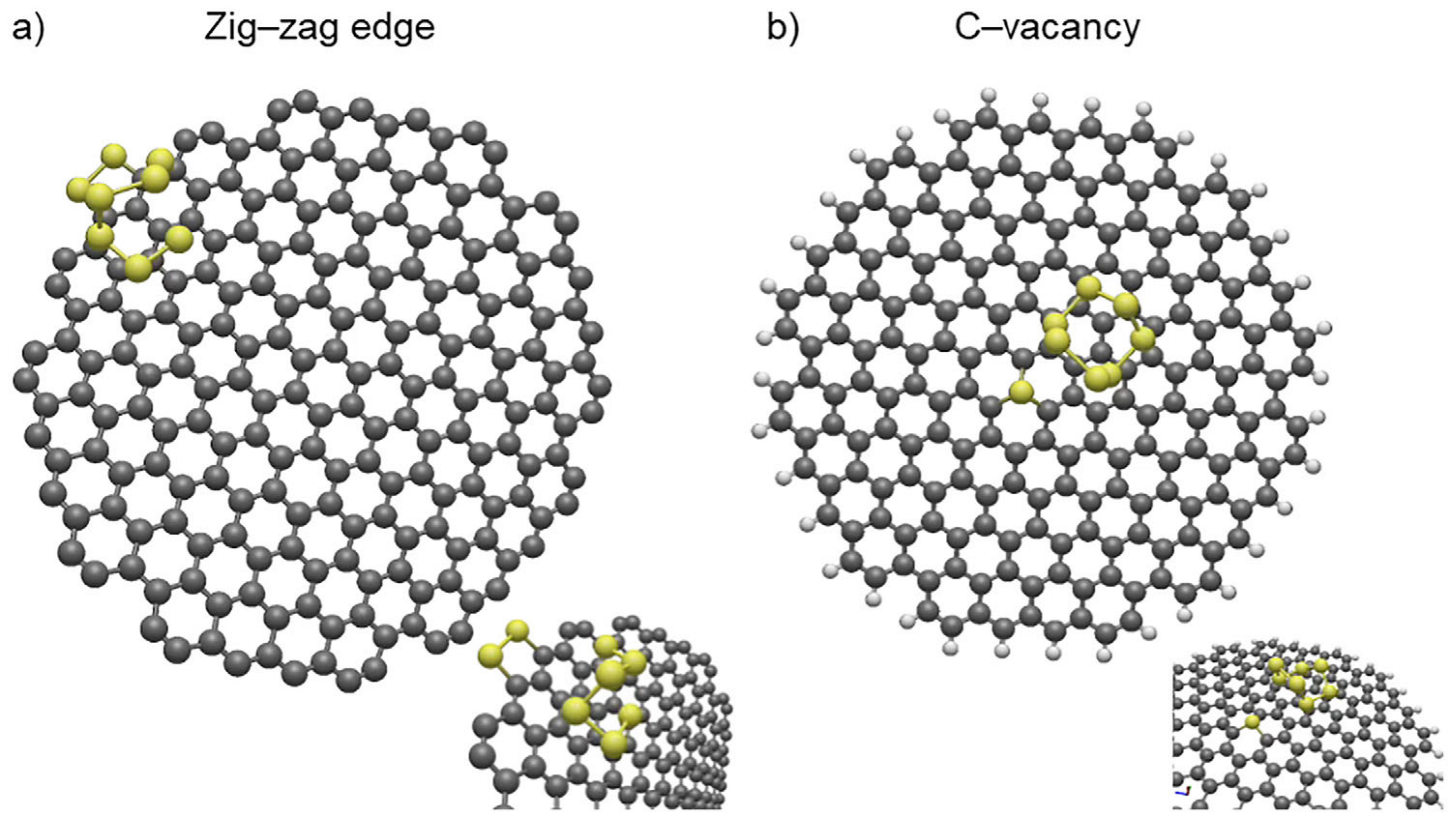
Final structures of graphene and sulfur after optimization when a reaction between carbon and sulfur occurs induced by a (a) zig‐zag edge or (b) C–vacancy.

Similarly, when the S_8_ molecule was placed next to the rim of the passivated graphene flake (in the same plane), no reaction occurred after optimization. Consequently, the hydrogen atoms were removed from the graphene flake, and the S_8_ molecule was placed in different distances perpendicular to the graphene flake and in different orientations. After optimization, the S_8_ molecule got cleaved, thus forming a C_3_S_8_ ring, which turned into a C_3_S_2_ ring with the remaining sulfur forming a free sulfur chain. This preferably occurred when the S_8_ molecule was placed next to a zig‐zag edge rather than an armchair edge (Figure 10). The most energetically‐favored configuration has a reaction energy of −5.58 eV (−538 kJ mol^−1^), which is in good agreement with the calculated value of −5.44 eV by Zhou et al. [27]. This energy was calculated by the difference between the absolute energy of the system at the final state after geometry optimization (−297908.58 eV) and the sum of the absolute initial energies of the S_8_ molecule (−86647.67 eV) and the graphene flake (−211255.33 eV). Further calculated adsorption energies are presented in Figure S7 and Table S1. The calculated reaction energy value is significantly higher than the reaction enthalpy obtained in the calorimetry, being expected since the experimental value serves as a lower limit and underestimates many contributions (see the Discussion above).

Introducing a single C‐vacancy in the graphene flake induces a reaction between carbon and sulfur as well (Figure 10). While one sulfur atom fills the vacancy, the rest of the sulfur atoms form a S_7_ ring. This reaction mechanism agrees with the molecular simulation by Beltran and Balbuena, who proposed a mechanism where unpaired electrons located at the carbon atoms act as strong nucleophiles, cleaving the sulfur‐sulfur bond and forming a strong C─S bond [28]. Subsequently, the remaining sulfur chain reacts in a similar fashion.

Although DFT calculations have their own limitations, and the complex, heterogeneous structure of porous carbons presents a significant challenge for theoretical modeling, the results nevertheless demonstrate that sulfur–carbon interactions can be either repulsive or attractive, depending on the specific configuration considered. Specific defects can induce a chemical reaction leading to ring‐opening. It remains unclear, however, whether the calculated, local reactions are the cause for the spillover or if this is an associated effect.

### 2.6 Electrochemical Studies

**FIGURE 11:**
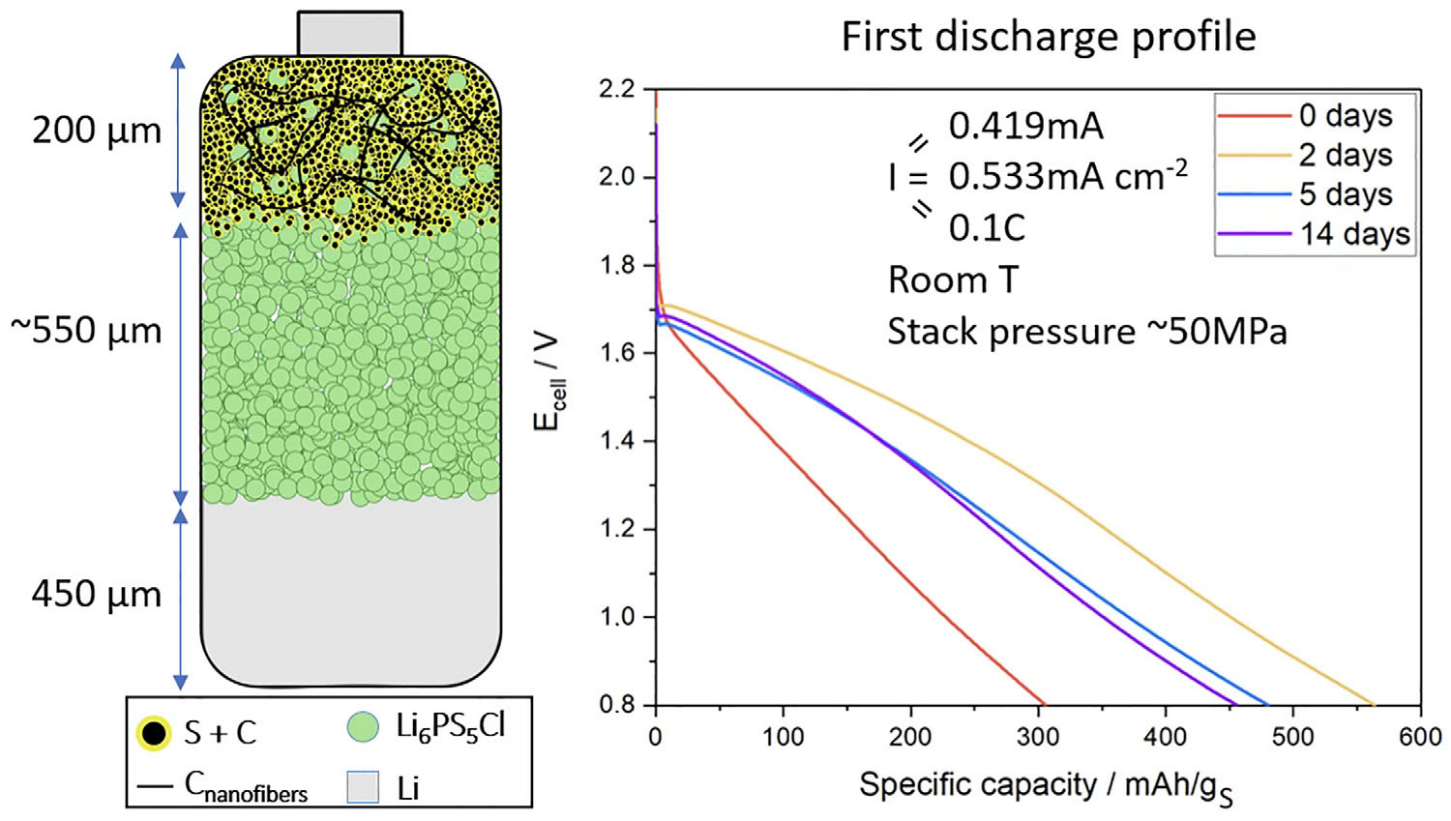
First discharge profiles for Li | Li_6_PS_5_Cl | (S [2.5 mg] + C [2.5 mg]) + C_nanofibers_ [1 mg] + Li_6_PS_5_Cl [4 mg] solid state batteries with different resting times for the S:C (1:1) mixture. The S:C mixture was prepared by mixing sulfur and carbon in an agate mortar by hand for 2 min. The cells were discharged at room temperature at C/10 under an initial stack pressure of 50 MPa. The capacity refers to the mass of sulfur.

The impact of the spillover in Li‐S batteries was studied qualitatively by assembling and cycling a series of identical solid‐state Li‐S batteries (Figure S8), differing only in the resting time of the sulfur‐carbon mixture prior to assembly. A solid‐state setup was chosen specifically to eliminate the possible influence of a liquid electrolyte adsorbed on the carbon. Limiting the cycling to the first discharge was chosen to minimize the impact of factors difficult to account, such as decomposition of the solid electrolyte, degradation of the interfaces, growth of dendrites, and mechanical stress. These factors can cause even apparently identical cells to deviate significantly from each other as the cycling progresses [29]. The cell that skipped a specific resting step and had only about two hours of rest between assembly and cycling provides a specific discharge capacity of about 300 mAh/g (Figure 11). Increasing the resting period to two days almost doubles the capacity, while even longer resting times actually reduce it to around 450 mAh/g. Further studies are required to clarify the cause of the decrease, but it can be hypothesized that after five days, a fraction of the sulfur had time to migrate to remote sites in the carbon structure, where it becomes disconnected from the solid electrolyte. This sulfur becomes thus electrochemically inactive and the overall capacity lower. After five days, the adsorption process is largely complete, which explains why the capacity is almost the same after 14 days. A more detailed investigation on the impact of sulfur spillover on the rechargeability and cycle life of Li–S batteries is under investigation.

## 3 Conclusions

This study expands the fundamental understanding of the sulfur spillover that arises when sulfur comes into contact with porous carbon. The pool of methods characterizing this process was expanded by means of microcalorimetry, SAXS, radiography as well as DFT calculations. For the first time, quantitative thermodynamic data was obtained. With microcalorimetry, it is found that the spillover process starts immediately when mixing both carbon and sulfur, and the lower limit of the net reaction enthalpy was determined as −28.2 kJ mol_S8_^−1^. The upper limit of the entropy change was calculated by analogy with a generic adsorption process and found to be 241.72 J mol^−1^ K^−1^. Using these values, the Gibbs free energy was estimated to be approximately −100.23 kJ mol_S8_^−1^. As the reaction is accompanied by a moderate enthalpy change and a strong entropy increase, and as the Tammann temperature of sulfur is significantly lower than ambient temperature, solid‐solid wetting cannot be excluded as the underlying process. At the same time, DFT calculations suggest reactions between S_8_ rings and defects (unpassivated edges or vacancies), including ring‐opening and formation of C─S bonds that could cause a further cascade of reactions. In view of the experimental and theoretical results, it is likely that the spillover process includes several different processes that occur simultaneously through the bulk sample mixture. The immediate onset of the spillover causes experimental challenges and, because porous carbons are thermodynamically poorly defined, a more rigorous analytical and theoretical treatment remains challenging to date.

The immediate onset of the spillover process may be critically important for metal‐sulfur batteries, which typically employ sulfur‐carbon composites as cathodes. Its impact is twofold, affecting both kinetics and thermodynamics. First, the spillover can introduce a time dependence in cathode activity. Second, the theoretical cell voltage of metal‐sulfur batteries is reduced by approximately 64 mV.

## 4 Experimental

### 4.1 S:C Preparation for SAXS and X‐Ray Radiography

The preparation of sulfur‐carbon (S:C) mixtures involved the utilization of sulfur powder (purity 99.5%, Sigma–Aldrich) and activated porous carbon (TF‐B520 sourced from MTI Corporation, subjected to pre‐treatment at 120°C under vacuum conditions for a duration of eight hours within an Argon‐filled glovebox). The activated carbon exhibited a BET surface area of 2,070 m^2^ g^−1^. Sulfur and carbon were manually mixed in a 1:1 weight ratio using a mortar inside an argon‐filled glovebox.

### 4.2 Small‐Angle X‐Ray Scattering (SAXS)

The dry S:C powder mixture was transferred into a cuvette with a 1 mm path length. The SAXS analysis was conducted using the SAXSess system (Anton Paar), which features a line‐focused beam employing monochromatic Cu‐Kα radiation (λ = 0.154 nm). This approach aimed to elucidate the structural characteristics of carbon powder specimens. The SAXS setup incorporated a one‐dimensional position‐sensitive detector (Mythen 2R, Dectris) to capture the scattering phenomena. We recorded the scattering profiles of the samples across a *q*‐vector range of 0.15 < *q* < 7 nm^−1^. The sample chamber was maintained at a reduced pressure of 0.5 mbar to mitigate potential contamination and minimize extraneous air scattering. Data smearing was rectified using the Strobl algorithm [30], noted for its enhanced numerical stability [31], and this process included comprehensive error propagation. Subsequent data adjustments accounted for variables such as sample transmission, background scattering, and detector sensitivity and were normalized against the primary flux. These corrections ensured the reliability of the data [32], and the final scattering curves were precisely calibrated to represent macroscopic scattering cross‐sections, expressed in units of cm^−1^.

### 4.3 X‐Ray Radiography

The X‐ray radiography [33] measurements were performed using a custom‐made µCT setup at the Helmoltz Zentrum Berlin [32]. The applied voltage of the tungsten source was 100 keV, and the current was set to 100 mA. The two images were taken 72 h apart using a flat panel detector and summing up image frames for 20 s with a resolution of approximately 10 mm per pixel. The images were dark field corrected and divided by the flat field by using the freely available open‐source software ImageJ.

### 4.4 Microcalorimetry

Pre‐dried elemental sulfur, activated carbon (AB520Y sourced from MTI Corporation, 1616 m^2^ g^−1^ (BET)) (Figure S9), carbon nanofibers (product nr. 719 781 from Sigma–Aldrich, 24 m^2^ g^−1^) and zeolithe (Carl Roth, 5 Å diameter, 600–700 m^2^ g^−1^) were mixed in the reported combinations in an agate mortar for 2 min (unless diversely indicated), transferred in sealed vials and then into a TAM IV microcalorimeter (TA Instruments). The measurements proceeded for several days, until the heat released approached ∼1 µW.

### 4.5 Solid‐State Cells

Solid‐state cells were assembled in an argon‐filled glovebox (MBRAUN). Sulfur and activated carbon (AB520Y from MTI Corporation, 1616 m^2^ g^−1^ (BET)) were mixed in a 1:1 weight ratio in an agate mortar for 2 min. To prepare the cathode, small amounts of this mixture were taken at different times (0 h, two days, five days, 14 days) and mixed in a Thinky Mixer (THINKY) with six zirconia balls (⌀ = 4 mm) at 2000 rpm for 1 min with carbon nanofibers and Li_6_PS_5_Cl (NEI Corporation) in a 5:1:4 weight ratio. The separator layer was prepared by pressing 70 mg of Li_6_PS_5_Cl directly into the cell (⌀ = 1 cm) at 400 MPa for 3 min. Above the separator layer were then spread 10 mg of the cathode composite and everything was pressed together at 400 MPa for 3 min. On the opposite side, a Li metal disk (⌀ = 0.8 cm, thickness = 0.45 mm) was added as the anode. The whole cell was then closed in a frame under ∼50 MPa of stack pressure (not compensated during cycling). The cells were connected to a BCS 805 (BioLogic) and discharged from OCV to 0.8 V at a current of 0.41875 mA (0.1 C) at room temperature.

### 4.6 DFT Calculations

Density functional theory calculations were performed using ORCA 6 [34, 35, 36, 37]. The calculations used a def2‐SVP basis set [38, 39]. The B3LYP functional [40, 41, 42, 43] was used to describe exchange‐ and correlation effects. To correctly describe the van‐der‐Waals interactions, the Grimme D3 dispersion correction [44] was employed. The charge of the studied system was 0, and the spin multiplicity was assigned as 1. All model structures were relaxed such that all atoms were allowed to move freely. Default parameters of ORCA were used to terminate the geometry relaxation.

## Author Contributions

**Román Healy Corominas**: validation, writing – review and editing. **Francesco Piccolo**: investigation, visualization, methodology, writing – review and editing, conceptualization. **Philipp Adelhelm**: funding acquisition, writing – review and editing, project administration, resources, supervision, conceptualization. **Jörg Schuster**: conceptualization, methodology, investigation. **Wilhelm Erth**: investigation, visualization. **Jesus Andrade**: investigation, visualization, writing – review and editing, methodology, software, data curation. **Marc Armbrüster**: funding acquisition, writing – review and editing, project administration, supervision, resources, conceptualization. **Anka Berger**: investigation, methodology, validation, writing – review and editing. **Sebastian Risse**: investigation, methodology, writing – review and editing, visualization, formal analysis, data curation. **Florian Fuchs**: investigation, methodology.

## Funding

This research was supported by Berlin Battery Lab, Humboldt‐University Berlin, Bundesanstalt für Materialforschung und ‐Prüfung (BAM) and Helmholtz‐Zentrum Berlin (HZB). The research was supported by the Deutsche Forschungsgemeinschaft (DFG, German Research Foundation) – project number 517014885.

## Conflicts of Interest

The authors declare no conflicts of interest.

## Supporting information




**Supporting File**: advs76931‐sup‐0001‐SuppMat.docx.

## Data Availability

Data are available upon reasonable request from the authors.
