# Supplementary material for: Further Evidences for the Sulfur Spillover on Carbon: Thermodynamic Aspects and Effects on Li‐S Solid‐State Batteries
Source: Adv Sci (Weinh). 2026 Aug 13:e76931. Online ahead of print. doi: 10.1002/advs.76931 (PMC13472108; doi:10.1002/advs.76931)
Supplement: Supplementary file 1 [file ADVS-9999-e76931-s001.docx]

**Further evidences for the sulfur spillover on carbon: thermodynamic aspects and effects on Li-S solid-state batteries**

*Francesco Piccolo^[a,b]^, Anka Berger^[c]^, Sebastian Risse^[b]^, Román Healy Corominas^[a]^, Wilhelm Erth^[d]^, Florian Fuchs^[e,f]^, Jesus Andrade^[g]^, Jörg Schuster^[e,f]^, Marc Armbrüster^[d]^, Philipp Adelhelm*^[a,b,h]^.*

*^a^Institute of Chemistry, Humboldt-Universität zu Berlin, Brook-Taylor-Str. 2, 12489 Berlin, Germany*

*^b^Research group operando battery analysis (CE-GOBA), Helmholtz-Zentrum Berlin für Materialien und Energie, Hahn-Meitner-Platz 1, 14109 Berlin, Germany*

^c^*BAM, Bundesanstalt für Materialforschung und -prüfung, Unter den Eichen 87, 12205 Berlin, Germany*

*^d^Inorganic Solid State and Material Chemistry, Eduard-Zintl-Institute, Technical University Darmstadt, 64287 Darmstadt, Germany*

*^e^Chemnitz University of Technology, Straße der Nationen 62, 09111 Chemnitz, Germany*

*^f^Fraunhofer Institute for Electronic Nano Systems, Technologie-Campus 3, 09126 Chemnitz, Germany*

*^g^Institute of Chemistry, Materials for Innovative Energy Concepts, Chemnitz University of Technology, Straße der Nationen 62, 09111 Chemnitz*

*^h^Center for the Science of Materials Berlin, Humboldt-Universität zu Berlin, Zum Großen Windkanal 2, 12489 Berlin, Germany*

**Figure S1**

Summary of the SAXS curve fitting of all 11 samples. The upper left corner displays all curves: pristine activated carbon (without sulfur) and 10 curves of activated carbon after mixing with sulfur for different durations. The coefficient of determination and the maximum value among the five fit parameters are reported for each curve to assess the model quality. Notably, the values remained below 10^−14^ for all parameters across all fits, indicating extremely high statistical significance. Furthermore, all *R^2^* values exceeded 0.9839, demonstrating excellent agreement between the model and the experimental SAXS data.

**SAXS analysis**

The intensity profile $I(q)$ of pristine and sulfur-loaded activated carbon (in cm^-1^) was modeled as the sum of three principal contributions:

$$I_{tot}\left( q \right)\text{=}I_{porod}\text{+}I_{mp}\text{+}I_{waxs}$$

*I_porod_* represents Porod scattering, caused by the sharp interfaces from macroscopic surface roughness;

*I_mp_* represents Teubner–Strey scattering, describing the microporous disordered network;

*I_waxs_* represent Broad WAXS features, notably two diffuse scattering maxima corresponding to the (002) and (100) reflections of carbon at scattering vectors $q_{p}$ of 17.6 nm⁻¹ ($d_{002}$ = 3.57Å) and 32.9 nm⁻¹ ($d_{100}$ = 1.9Å). The (002) reflection originates from the average interlayer distance between turbostratically stacked graphene layers, while the (100) reflection reflects in-plane correlations within disordered graphitic domains. In highly disordered carbons—such as activated or hard carbon—these peaks are inherently broad and asymmetric, due to pronounced structural disorder including layer curvature, stacking faults, and limited crystallite coherence lengths.$L(q)$To model these features, both reflections were described using pure Lorentzian functions $A_{0}$ with amplitude $q_{p}$, centered at $\gamma$, and a characteristic width ￼, capturing the intrinsic disorder-broadened nature of the signal. No Gaussian convolution was applied, as instrumental resolution effects are negligible compared to the extensive intrinsic broadening. This simplification avoids overfitting and is consistent with their origin in strongly curved or crumpled turbostratic graphene domains, rather than well-defined crystallites.$1\text{/}q^{2}$In addition, a $I_{waxs}$ decay term was explicitly introduced into the WAXS component $q$ to accurately capture the intensity behavior in the intermediate-to-high $q^{\text{-}4}$-range. This correction is crucial because, unlike ideal systems where the SAXS intensity falls off with ￼ according to Porod’s law, the measured SAXS profiles for disordered carbons often exhibit a slower decay, especially in the region dominated by ￼^1^$q^{\text{-}2}$^]^, such $q^{\text{-}2}$ behavior arises from the orientation-averaged Lorentzian broadening of reflections associated with curved or crumpled graphene layers. This effect not only extends into the SAXS regime but also reflects a mass-fractal-like topology that bridges atomic-scale ordering with mesoscale pore morphology.

Incorporating the $1\text{/}q^{2}$ factor ensures continuity between the WAXS and SAXS regimes and provides a physically meaningful description of the scattering intensity over the full measured $q$-range. This hybrid approach enables the model to account for both the microstructural peak broadening and the underlying structural topology of disordered carbon matrices.

$$L\left( q \right)\text{=}\frac{A_{0}}{q^{2}}\cdot\frac{1}{\pi\left( q_{p}^{2}\text{-}q^{2} \right)\text{+}\gamma^{2}}$$

The Teubner–Strey model^[2]^ is especially suitable for disordered two-phase systems like microporous carbons, as it describes scattering from domains with short-range order but without periodicity. The intensity function is expressed as:

$$L\left( q \right)\text{=}\frac{A_{0}}{q^{2}}\frac{1}{\pi\left( q\frac{2}{p}\text{-}q^{2} \right)\text{+}\gamma^{2}}$$

From this, several physically relevant quantities can be derived:

- The correlation length $\xi$, which quantifies the spatial extent of pore ordering:

$$\xi\text{=}\left( \frac{1}{2\sqrt{C_{2}}}\text{+}\frac{C_{1}}{4C_{2}} \right)^{\text{-}\frac{1}{2}}$$

- The specific micropore surface area *S_mp_*, which relates to *I_0_* and *C_2_* as:

$$S_{mp}\text{=}\frac{I_{0}}{2\pi C_{2}\left( \Delta SLD \right)^{2}}\Rightarrow rel.S_{mp}\text{=}\frac{\frac{I_{0}}{I_{0}\left( AC \right)}}{\frac{C_{2}}{C_{2}\left( AC \right)}}$$

*S_mp_* could not be calculated in absolute units, as the scattering length density contrast *ΔSLD* between the carbon matrix and the pore space is not precisely known for this system. To enable a relative comparison between samples, we therefore normalized all calculated *S_mp_* values to the value obtained for the pristine activated carbon (AC) sample without sulfur. This approach provides a consistent basis for quantifying changes in microporosity upon sulfur incorporation, even in the absence of absolute contrast calibration.

In addition, the Porod constant—reflecting the asymptotic behavior of the scattering intensity in the high-$q$ regime—can be extracted from the low-angle scattering region (typically following a $q^{\text{-}4}$ dependence). While the absolute Porod surface area also depends on $\Delta SLD$, the relative trends of the Porod contribution can provide further insight into changes in macroscopic surface area and interface sharpness among samples. Together with the Teubner–Strey parameters, the Porod analysis offers a comprehensive view of both the micro- and mesoscale structural evolution in the disordered carbon system.

The Teubner–Strey model was justified for our system based on the smooth and broad hump visible in the intermediate *q*-region, consistent with a sponge-like micropore network exhibiting slight short-range ordering-behavior that deviates from both ideal globular and fractal aggregates. This agrees with previous literature, including the work of Saurel *et al.*^[1]^ who used the same formalism to model SAXS curves from a range of disordered carbonaceous materials.

As shown in Figure 3 of the manuscript, the WAXS contribution to the SAXS intensity is predominantly governed by the *q*^-2^ behavior arising from the (002) reflection. Consequently, only this peak was included in the *I(q)* fitting function, and a scaling factor $K$ was introduced to account for changes in the scattering length density contrast *ΔSLD* due to sulfur infiltration. The peak position *q*_002_ and the peak width $\gamma_{002}$ obtained from the fit in Figure 3, were held constant throughout the analysis.

## Figure S2

Heat evolution of mixtures with different S:C ratios at 25°C. In the zoomed-in layer, continuous lines correspond to the compositions with a carbon excess (ratios from 0.1:1 to 0.8:1), the dashed grey line to the ratio 1:1 and the dotted lines to the compositions with a sulfur excess (ratios from 1.5:1 to 4:1). The ratios close to 1:1 gave the strongest heat, suggesting S and C react in a similar ratio. The data was normalized according to the total weight of the sample (250 mg for ratios from 0.1:1 to 1:1, 400 mg for ratios from 1.5:1 to 4:1). The measurements started approximately 1h after the preparation of the mixtures (t = 0).

## Figure S3

Reference measurements for the elements. The graph shows the heat evolution at 25°C for samples containing only elemental sulfur (yellow), elemental carbon (black) and elemental carbon after 2 minutes of grinding in a mortar (red). The heat flow is normalized to the weight of the sample (250 mg in all cases). Differently from the S:C mixtures, the heat flow is negligible.

## Figure S4: Reproducibility test microcalorimetry

Reproducibility test: the heat released by 3 different S:C ratios was measured twice in two different experiments. The resulting curves overlap almost perfectly, with the minor deviations probably caused by the slightly different weighted amounts and times between mixing and measuring. The data was normalized according to the total weight of the sample.

## Figure S5

Heat evolution at 60°C of mixtures of sulfur with activated carbon (red), carbon nanofibers (black) or zeolite (yellow) with the same S:X ratio (2:1). Only the mixture of sulfur with activated carbon released significant heat, confirming the phenomenon happens only when both a high surface area and a carbon-based composition are present in the substrate. As expected, the reaction proceeds even faster than at room temperature, resulting close to complete in just a few hours. The data was normalized according to the total weight of the sample.

## Figure S6

Distribution measured by EDX of sulfur (in pink) in samples of S:C in 1:1 weight ratio mixed for a) 1 minute, b) 2 minutes and c) 4 minutes. Longer times were avoided to limit heating the materials by friction in the mortar. d) Heat evolution at 25°C of S:C 1:1 mixture mortared for 1, 2 and 4 minutes. The data was normalized according to the total weight of the sample (250 mg). While increasing the mixing time improves the homogeneity of the sample, the deviations between their heat signals are minimal.

**Figure S7**

**With spin-polarization**

**Without spin-polarization**

Results of the DFT calculations performed with and without spin-polarization. For each case:
Top row: final structures after geometric relaxation of sulfur attached to unpassivated graphene flakes at armchair-edges (a, b and c) or zigzag-edges (d, e, f). Bottom: calculated adsorption energies of a S_8_ molecule on an unpassivated graphene flake.

Before the geometry relaxation, the S_8_ molecule was located on different positions: on a zigzag-edge or an armchair-edge and shifted in the out-of-plane direction by Δ. Furthermore, the S_8_ molecule was either placed parallel to the graphene flake or rotated by 90°. Finding the configuration with the lowest energy (global minimum) is difficult using density functional theory, as the system tends to get stuck in local minima. Consequently, all the relaxations lead to a different final structure, each corresponding to a different local minimum in the energy landscape. In reality, thermal fluctuation would lead to transitions between those local energy minima. We have performed calculations with and without spin-polarization and report the results for each of these methods.

From Figure S7 it can be observed that structures with a closed S_8_ ring have a higher energy than those with an open S_8_ ring (compare structure (a) and (b), independent on whether spin-polarization was considered). The minimal values for the zigzag-edge are comparable to the calculated value of -5.44 eV, which was reported by Zhou *et al.*^[3]^. Focusing on the structures with a very low energy (e.g., structures (d), (e), and (f)), it can be seen that the structures differ, but they all show a strong interaction between the S_8_ and graphene. This clearly supports the conclusion that the S_8_ molecule reacts with the graphene flake at unpassivated edges. Again, this is independent on whether spin-polarization was considered or not.

Summing up the comparison with and without spin-polarization, we note that there are differences in the final structure after the relaxation as well as the precise adsorption energies, indicating the open-shell character for some of the structures. The general conclusions regarding the interaction of the S_8_ with the graphene, however, are comparable.

The calculated energy values are summarized in the following table.

**Table S1: Calculated energy values of different structures**

| **Edge type** | **Shift [Å]** | **Rotation [°]** | **Energy [eV] (without spin-polarization)** | **Energy [kJ/mol] (without spin-polarization)** | **Energy [eV] (with spin-polarization)** | **Energy [kJ/mol] (with spin-polarization)** |
| --- | --- | --- | --- | --- | --- | --- |
| armchair | -1 | 0 | -3.83342 | -369.874 | -4.23092 | -408.228 |
| armchair | -1 | 90 | -0.70519 | -68.0421 | -0.58876 | -56.8073 |
| armchair | -2 | 0 | -1.54839 | -149.4 | -0.42674 | -41.1748 |
| armchair | -2 | 90 | -1.03936 | -100.285 | -0.63766 | -61.5262 |
| armchair | 0 | 0 | -3.06598 | -295.827 | -4.22447 | -407.606 |
| armchair | 0 | 90 | -0.99184 | -95.6994 | -0.59575 | -57.4821 |
| armchair | 1 | 0 | -4.67774 | -451.341 | -4.23245 | -408.376 |
| armchair | 1 | 90 | -1.0175 | -98.1751 | -0.60299 | -58.1803 |
| armchair | 2 | 0 | -1.52677 | -147.313 | -0.41861 | -40.3906 |
| armchair | 2 | 90 | -1.03128 | -99.5052 | -0.61113 | -58.9656 |
| zigzag | -1 | 0 | -4.84366 | -467.35 | -4.12437 | -397.948 |
| zigzag | -1 | 90 | -6.27978 | -605.916 | -5.23508 | -505.116 |
| zigzag | -2 | 0 | -3.19774 | -308.54 | -4.08807 | -394.445 |
| zigzag | -2 | 90 | -5.45329 | -526.171 | -4.2058 | -405.804 |
| zigzag | 0 | 0 | -6.24405 | -602.469 | -4.143 | -399.745 |
| zigzag | 0 | 90 | -6.13958 | -592.389 | -4.53213 | -437.291 |
| zigzag | 1 | 0 | -6.09903 | -588.477 | -4.10417 | -395.998 |
| zigzag | 1 | 90 | -5.05141 | -487.394 | -4.47039 | -431.334 |
| zigzag | 2 | 0 | -5.96733 | -575.769 | -3.18336 | -307.152 |
| zigzag | 2 | 90 | -6.18143 | -596.427 | -3.73216 | -360.104 |

The calculations were performed using QuantumATK W-2024.09-SP2^[4,5]^. The PBE functional has been used to describe exchange- and correlation effects^6^. Numerical basis sets are used by QuantumATK, where we have used the ones with accuracy “high”. The atomic coordinates have been relaxed until the forces were below 0.01 eV/Å.

## Figure S8: Solid-state batteries fabrication steps

Sulfur and activated carbon were mixed in a 1:1 weight ratio in an agate mortar for 2 minutes. To prepare the cathode, small amounts of this mixture were taken at different times (0 hours, 2 days, 5 days, 14 days) and mixed in a Thinky Mixer (THINKY) with 6 zirconia balls (⌀=4 mm) at 2000 rpm for 1 min with carbon nanofibers and Li_6_PS_5_Cl (NEI Corporation) in a 5:1:4 weight ratio. The separator layer was prepared by pressing 70 mg of Li_6_PS_5_Cl directly into the cell (⌀=1 cm) at 400 MPa for 3 minutes. Above the separator layer were then spread 10 mg of the cathode composite and everything was pressed together at 400 MPa for 3 minutes. On the opposite side, a Li metal disk (⌀=0.8 cm, thickness=0.45 mm) was added as the anode. The whole cell was then closed in a frame under ~50 MPa of stack pressure (not compensated during cycling). The cells were connected to a BCS 805 (BioLogic) and discharged from OCV to 0.8 V at a current of 0.41875 mA (0.1 C) at room temperature.

## Figure S9: Nitrogen physisorption microcalorimetry carbon

Nitrogen physisorption measurements of the commercial activated carbon used for microcalorimetry. Left: N_2_ (77.4 K) adsorption–desorption isotherm. *A_BET_ =* 1616 m^2^/g. Right: pore size distribution (four peaks at values of 0.58, 0.82, 1.14 and 2.19 nm).

## Figure S10: Nitrogen physisorption SAXS and X-ray radiography carbon

Nitrogen physisorption measurements of the commercial activated carbon used for SAXS and X-ray radiography. Reproduced with permission from Medenbach et al.^[6]^ a) BET isotherms for the porous carbon with and without sulfur (mass ratio 1:1). b) Cumulative pore volume as a function of pore width (DFT method). *A_BET_ =* 2,070 m^2^ g^-1^.

**Table S2: Pore size distribution of the SAXS and X-ray radiography carbon**

| Pore width / nm | Pore volume / cm³ g^-1^ |
| --- | --- |
| 0 – 1  1 – 2  2 – 5  5 – 10  10 - 20 | 0.285  0.426  0.150  <0.01  <0.01 |

Pore size distribution of the porous carbon used for SAXS and X-ray radiography measured with DFT method as reported by Medenbach et al.^[6]^

**Differences between the two carbon materials**

Both carbons are highly porous with BET surface areas exceeding 1600 m^2^ g^-1^. The reason for using different carbons is that one carbon became unavailable during the course of the study and it was not possible to conduct another long-term measurement (SAXS over 29 days) due to limited instrumental access. Medenbach et al.[6] showed that the spillover capacity depends on the specific surface area. For the SAXS and radiography measurements, the loading is clearly below the spillover capacity, ensuring that the complete spillover effect can be captured eventually. Differences in surface chemistry may impact the enthalpy of the reaction but given the high porosity of both carbons, we do not expect any major difference. This is also supported by the fact that cell voltages for Li-S batteries (either with liquid or solid electrolyte) provide similar cell voltages irrespective of the type of carbon. Overall, while the carbon surface chemistry will impact the sulfur-carbon interaction, the difference in enthalpy is likely small.

**References**

(1)	Saurel, D.; Ségalini, J.; Jáuregui, M.; Pendashteh, A.; Daffos, B.; Simon, P.; Casas-Cabanas, M. Corrigendum to “A SAXS Outlook on Disordered Carbonaceous Materials for Electrochemical Energy Storage” [Energy Storage Mater. 21 (2019) 162–173]. *Energy Storage Materials* **2020**, *28*, 418. <https://doi.org/10.1016/j.ensm.2020.03.013>.

(2)	Teubner, M.; Strey, R. Origin of the Scattering Peak in Microemulsions. *The Journal of Chemical Physics* **1987**, *87* (5), 3195–3200. <https://doi.org/10.1063/1.453006>.

(3)	Zhou, G.; Yang, A.; Gao, G.; Yu, X.; Xu, J.; Liu, C.; Ye, Y.; Pei, A.; Wu, Y.; Peng, Y.; Li, Y.; Liang, Z.; Liu, K.; Wang, L.-W.; Cui, Y. Supercooled Liquid Sulfur Maintained in Three-Dimensional Current Collector for High-Performance Li-S Batteries. *Science Advances* **2020**, *6* (21), eaay5098. <https://doi.org/10.1126/sciadv.aay5098>.

(4)	Smidstrup, S.; Markussen, T.; Vancraeyveld, P.; Wellendorff, J.; Schneider, J.; Gunst, T.; Verstichel, B.; Stradi, D.; Khomyakov, P. A.; Vej-Hansen, U. G.; Lee, M.-E.; Chill, S. T.; Rasmussen, F.; Penazzi, G.; Corsetti, F.; Ojanperä, A.; Jensen, K.; Palsgaard, M. L. N.; Martinez, U.; Blom, A.; Brandbyge, M.; Stokbro, K. QuantumATK: An Integrated Platform of Electronic and Atomic-Scale Modelling Tools. *J. Phys.: Condens. Matter* **2019**, *32* (1), 015901. <https://doi.org/10.1088/1361-648X/ab4007>.

(5)	van Setten, M. J.; Giantomassi, M.; Bousquet, E.; Verstraete, M. J.; Hamann, D. R.; Gonze, X.; Rignanese, G.-M. The PseudoDojo: Training and Grading a 85 Element Optimized Norm-Conserving Pseudopotential Table. *Computer Physics Communications* **2018**, *226*, 39–54. <https://doi.org/10.1016/j.cpc.2018.01.012>.

(6)Medenbach, L.; Escher, I.; Köwitsch, N.; Armbrüster, M.; Zedler, L.; Dietzek, B.; Adelhelm, P. Sulfur Spillover on Carbon Materials and Possible Impacts on Metal–Sulfur Batteries. *Angewandte Chemie International Edition* **2018**, *57* (41), 13666–13670. <https://doi.org/10.1002/anie.201807295>.
